# Long-term medical and productivity costs of severe trauma: Results from a prospective cohort study

**DOI:** 10.1371/journal.pone.0252673

**Published:** 2021-06-04

**Authors:** Marjolein van der Vlegel, Juanita A. Haagsma, Roos J. M. Havermans, Leonie de Munter, Mariska A. C. de Jongh, Suzanne Polinder

**Affiliations:** 1 Department of Public Health, Erasmus MC, University Medical Center Rotterdam, Rotterdam, The Netherlands; 2 Brabant Trauma Registry, Network Emergency Care Brabant, Tilburg, The Netherlands; 3 Department Trauma TopCare, ETZ Hospital, Tilburg, The Netherlands; University Hospital Zurich, SWITZERLAND

## Abstract

**Background:**

Through improvements in trauma care there has been a decline in injury mortality, as more people survive severe trauma. Patients who survive severe trauma are at risk of long-term disabilities which may place a high economic burden on society. The purpose of this study was to estimate the health care and productivity costs of severe trauma patients up to 24 months after sustaining the injury. Furthermore, we investigated the impact of injury severity level on health care utilization and costs and determined predictors for health care and productivity costs.

**Methods:**

This prospective cohort study included adult trauma patients with severe injury (ISS≥16). Data on in-hospital health care use, 24-month post-hospital health care use and productivity loss were obtained from hospital registry data and collected with the iMTA Medical Consumption and Productivity Cost Questionnaire. The questionnaires were completed 1 week and 1, 3, 6, 12 and 24 months after injury. Log-linked gamma generalized linear models were used to investigate the drivers of health care and productivity costs.

**Results:**

In total, 174 severe injury patients were included in this study. The median age of participants was 55 years and the majority were male (66.1%). The mean hospital stay was 14.2 (SD = 13.5) days. Patients with paid employment returned to work 21 weeks after injury. In total, the mean costs per patient were €24,760 with in-hospital costs of €11,930, post-hospital costs of €7,770 and productivity costs of €8,800. Having an ISS ≥25 and lower health status were predictors of high health care costs and male sex was associated with higher productivity costs.

**Conclusions:**

Both health care and productivity costs increased with injury severity, although large differences were observed between patients. It is important for decision-makers to consider not only in-hospital health care utilization but also the long-term consequences and associated costs related to rehabilitation and productivity loss.

## Introduction

Injury is a leading cause of death and disability for both young and older adults [1]. In 2017, 5% of the registered trauma patients in the Netherlands were severely injured (Injury Severity Score (ISS) ≥ 16) [2]. The last decades, there has been a decline in injury mortality through improvements in trauma care [3, 4]. Subsequently, the focus on non-fatal outcome has become increasingly important [5]. People who survive a severe trauma are at risk of both short-term and long-term disabilities [6–10]. Severe injury has a large impact on health and leads to a substantial decrease of health-related quality of life [11–15] and loss of productive work-years [16, 17]. In a previous Dutch study including severely injured patients, 60% returned to their pre-injury work status within two years after trauma [17].

Apart from the burden on a person’s life, severe trauma is also putting a high burden on society regarding health care utilization and health care costs related to severe trauma. Although multiple studies have explored long-term consequences of trauma on health-related quality of life [6–10], few studies have investigated long-term economic consequences of trauma. Several studies have estimated the costs of severe trauma, however, these studies were mainly focused on short-term hospital care [18]. The studies that did provide information on long-term costs of injury were mainly focused on specific injury types or focused on the entire trauma population, consisting of mainly minor injuries [19–24]. A societal perspective, considering long-term care and productivity loss can result in a more complete overview of the costs of severe injuries.

The assessment of the health care utilization and productivity loss and associated health care and productivity costs provides insight into the economic burden of severe trauma on society. In this study we aimed to determine the total long-term costs of severe trauma patients. We provide a detailed overview of health care utilization, total in-hospital and post-hospital medical costs and productivity costs of severe trauma patients up to 24 months after sustaining injury. We investigated the impact of injury severity level on health care utilization and costs and determined predictors for health care and productivity costs.

## Methods

### Study population

This prospective cohort study, including severely injured patients, is part of the Brabant Injury Outcome Surveillance (BIOS) study [25]. Severely injured patients are defined as patients with an injury severity score (ISS) ≥ 16. Adult injury patients (≥ 18 years) who were admitted to a hospital ward or Intensive Care Unit (ICU) in the region Noord-Brabant, the Netherlands, between August 2015 and November 2016, and who survived hospital discharge, were eligible for inclusion in the BIOS study. Exclusion criteria were pathological fractures, insufficient knowledge of the Dutch language and the absence of a permanent address. Eligible patients were included in this study if they completed at least one questionnaire and length of stay at a hospital ward or ICU was available. A proxy informant completed the self-reported questionnaires if patients were incapable of completing the questionnaire themselves. All participants and proxy informants signed an informed consent for participation in the BIOS study. The BIOS study has been approved by the Medical Ethics Committee Brabant (NL50258.028.14). A detailed description of the costs calculations can be found in a previous study describing the health care and productivity costs for the entire trauma population [23].

### Patient and injury characteristics

During a follow-up of 24 months, data was collected with repeated questionnaires at 1 week and 1, 3, 6, 12 and 24 months after injury. The first questionnaire included questions on sociodemographic characteristics including age, sex, comorbidities and health status. Health status was measured with the EQ-5D-3L at 1 week after injury. If patients did not fill in the 1 week questionnaire, the score from 1 month questionnaire was used. The Dutch value set was used to calculate the utility score ranging from 0 (death) to 1 (full health). The utility score can also have a negative value for health states worse than death [26].

Data on type of injury, mechanism of trauma (e.g. falls, traffic) and Abbreviated Injury Scale (AIS)-codes were obtained from the Brabant Trauma Registry (BTR). AIS coding was done by personnel or medical professionals who received a training in injury coding. Each injury is attributed an Abbreviated Injury Scale (AIS) score (AIS-90, update 2008), allocated to one of six body regions [27]. The ISS is obtained by taking the sum of the squares of the AIS scores of the three most severely injured body regions and was automatically calculated based on the AIS scores that were registered in the Brabant Trauma Registry [28]. With the ISS the overall trauma severity is assessed. ISS ranges from 1 to 75. Major trauma is considered when ISS > 15. In this study, ISS was categorized as severe (16–24), and profound (≥25) [29].

### Health care use and productivity loss

In-hospital medical procedures (e.g. emergency department visit, diagnostics, admission to ICU and transport to the hospital) are obtained from the Brabant Trauma registry. Detailed information on intramural and extramural care are collected with the iMTA Medical Consumption Questionnaire (iMCQ), a self-reported questionnaire with questions related to non-disease specific healthcare consumption [30]. The iMCQ assesses whether patients had an appointment with various health care professionals, both intramural (e.g. stay or treatment at a medical facility) and extramural (e.g. homecare). Information on productivity loss at work was collected with the iMTA productivity cost questionnaire (iPCQ) [31], consisting of questions on both the absence of work due to injury and on being present at work after injury but being less productive than before the injury. The iMCQ and iPCQ were included in all questionnaires at 1, 3, 6, 12 and 24 months after injury.

### Health care costs

All unit costs were retrieved from a cost-reference manual [32], presented in S1 Table except for unit costs of diagnostics, which were retrieved from hospital price lists, previous research and the Dutch Healthcare Authority (NZa) [33–40]. Healthcare costs were calculated by multiplying healthcare use per period with cost per unit. All costs are expressed in 2017 Euros. Health care costs per service was presented for the group of respondents with available health care consumption data, since not all respondents filled out every question in the questionnaire. Health care costs were divided in in-hospital costs and post-hospital costs. In-hospital costs consisted of costs for: transportation to the emergency department (ED), stay at a hospital ward, stay at an intensive care unit (ICU) and diagnostics. Post-hospital health care costs consisted of the costs of: stay in an institution (e.g. rehabilitation centre), homecare and visits to practitioners (e.g. general practitioner, psychologist, physiotherapist). In the Netherlands, both hospitalization and long-term medical care are covered by a mandatory insurance.

### Productivity costs

The productivity costs were determined with the friction costs method. The friction costs method assumes that productivity loss is confined to the period needed to find replacement for the absent employee. We used a friction period of 85 days, in accordance with Dutch guidelines. Productivity loss longer than the friction period was valued equal to 85 days [32]. If the number of hours a participant worked per week were missing, the national mean by sex was used, 36 hours per week for males and 26 hours per week for females [41]. The costs of productivity loss were determined by multiplying the total number of hours of work missed with the hourly wage rate. The average wage rates per sex can be found in S1 Table. The productivity costs were determined for the working population (patients aged 18–67).

### Statistical analysis

Pearson’s chi-square, Fisher’s exact test and Mann-Whitney U test were used for comparisons between participants and non-participants. Descriptive statistics (mean, standard deviation (SD), median, and interquartile range (IQR)) were used to determine the health care utilization, in-hospital costs, post-hospital costs and productivity costs for patients with ISS 16–24 (severe) and ISS ≥25 (profound). Health care and productivity costs were rounded to the nearest 10 euro. An AIS score of ≥3 was considered as severe injured body region. ‘Head injury’ included head, face and neck injuries. ‘Extremity injury’ included upper and lower extremity injuries. ‘Thorax injury’ included thorax and abdomen injuries Predictors for health care costs and productivity costs were assessed with log-linked gamma generalized linear models (GLM). The relative difference in mean costs (exp[parameter estimate]) with 95% Confidence Interval (CI) were reported. For the GLM, only patients with available data on patient and injury characteristics and costs were included. Patients who died during the two-year follow-up were not included in our analysis. All statistical analyses were performed in IBM SPSS version 24 (Armonk, NY: IBM Corp, USA). A p-value of ≤0.05 was considered statistically significant.

## Results

### Sociodemographic and injury characteristics

A total of 433 severely injured patients were asked to participate in this study and 239 (55.2%) signed an informed consent. There were 174 (40.2%) severe injury patients who completed the iMCQ and iPCQ (Table 1). Of these, 115 (66.1%) patients were male. The median age was 55 years and the majority of injuries were caused by traffic collisions (n = 74, 42.5%) and home and leisure accidents (n = 73, 42.0%). The median ISS was 20 (IQR: 17–24) and 43 (24.7%) patients had an ISS of 25 or higher. Head injuries (n = 77, 44.3%) and thorax injuries (n = 75, 43.1%) were most common and most patients had injuries in one body region (n = 124, 71.3%). Compared to non-participants, participants were significantly younger, had a higher ISS and had more patients with injuries in multiple body regions. Head injuries were less common in participants and lower extremity injuries were more common, compared to non-participants.

**Table 1:** Characteristics of the study population, by participation.

	Participants	Non-participants	P-value
**Participants, n**	174	259	
**Sex, n (%)**			0.45
Female	59 (33.9)	97 (37.5)	
Male	115 (66.1)	162 (62.5)	
**Age in years, median (IQR)**	55 (40–66)	61 (43–74)	0.01
**Age group, n (%)**			
18–44 years	49 (28.2)	67 (25.9)	
45–67 years	86 (49.4)	98 (37.8)	
≥68 years	39 (22.4)	94 (36.3)	
**External cause, n (%)**^b^			0.09
Traffic	74 (42.5)	96 (37.1)	
Home and Leisure	73 (42.0)	102 (39.4)	
Occupational	14 (8.0)	8 (3.1)	
Sport	8 (4.6)	8 (3.1)	
Intentional	4 (2.3)	15 (5.8)	
**ISS, median (IQR)**	20 (17–24)	19 (17–22)	0.04
**ISS, n (%)**			
16–24	131 (75.3)	201 (77.6)	
≥25	43 (24.7)	58 (22.4)	
**Injured body region (AIS ≥3), n (%)**^a^^c^			
Head	77 (44.3)	142 (54.8)	0.03
Thorax	75 (43.1)	98 (37.8)	0.27
Abdomen	19 (10.9)	22 (8.5)	0.40
Spine	23 (13.2)	26 (10.0)	0.31
Upper extremity	3 (1.7)	0 (0)	0.06
Lower extremity	40 (23.0)	34 (13.1)	0.01
**Number of injured body regions, n (%)**^c^			0.03
1	124 (71.3)	187 (72.2)	
2	38 (21.8)	61 (23.6)	
≥3	11 (6.3)	4 (1.5)	
**Comorbidity status, n (%)**^d^			
No comorbidity	86 (49.4)	n.a.	
≥1 comorbidities	83 (47.7)	n.a.	
**EQ-5D utility score, mean (SD)**^e^	0.42 (0.34)	n.a.	

ISS = Injury Severity Score TBI = Traumatic Brain Injury, AIS = Abbreviated Injury Scale, SD = standard deviation, n.a. = not available.

^a^ Percent of total adds up to more than 100% because patients can have multiple injuries and injuries in multiple body regions.

^b^ 1 (0.6%) missing value for participants and 30 (11.6%) missing values for non-participants.

^c^ 1 (0.6%) missing value for participants and 7 (2.7%) missing values for non-participants.

^d^ 5 (2.9%) missing values.

^e^ Indicating health status on a scale 0 (death) -1 (full health), 26 (14.9%) missing values.

### Health care utilization

A detailed overview of health care utilization is shown in Table 2. On average, patients stayed at a hospital ward for 14.2 (SD: 13.5) days. Of patients with ISS 16–24, 61.1% stayed at an ICU and 97.7% of patients with ISS ≥25. After hospital discharge, 19.3% of patients with ISS 16–24 and 45.5% of patients with ISS ≥25 received day treatments at a rehabilitation center, with respectively 25.4 (SD: 21.9) and 33.3 (SD: 27.3) visits. The majority of patients received physical therapy (72.0%), with a mean of 51.6 (SD:54.7) visits. Patients with paid employment (n = 89,51.1%) returned to work on average 21.0 (SD: 22.7) weeks after hospital admission.

**Table 2:** Health care utilization by category of service of adult trauma patients (ISS≥16).

	Total (n = 174)		ISS 16–24 (n = 131)		ISS≥25 (n = 43)	
Health care utilization	N (%)	Mean (SD) ^a^	N (%)	Mean (SD) ^a^	N (%)	Mean (SD) ^a^
Transport						
*Road ambulance*	134 (77.0%)		106 (80.9%)		28 (65.1%)	
*Road ambulance with assistance of HEMS*	28 (16.1%)		14 (10.7%)		14 (32.6%)	
*Private transport*	12 (6.9%)		11 (8.4%)		1 (2.3%)	
Stay at a hospital ward, days	174 (100%)	14.2 (13.5)	131 (100%)	11.7 (9.9)	43 (100%)	21.7 (19.3)
Stay at the ICU, days	122 (70.1%)	2.8 (1.2)	80 (61.1%)	2.5 (1.0)	42 (97.7%)	3.4 (1.2)
Stay at a nursing home, days	7 (4.0%)	56.7 (36.9)	7 (5.3%)	56.7 (36.9)	0 (0.0%)	-
Stay at a rehabilitation center, days	15 (8.6%)	39.7 (25.3)	10 (7.6%)	26.6 (16.2)	5 (11.6%)	66.0 (18.9)
Day treatment at a rehabilitation center, visits	37 (25.2%)	28.5 (24.1)	22 (19.3%)	25.4 (21.9)	15 (45.5%)	33.3 (27.3)
General practitioner, visits	104 (63.8%)	4.2 (3.8)	74 (60.2%)	3.7 (3.3)	30 (75.0%)	5.5 (4.6)
Occupational health physician/therapist, visits	85 (52.1%)	8.3 (10.4)	61 (49.6%)	6.9 (8.7)	24 (60.0%)	11.9 (13.5)
Psychologist, visits	45 (28.3%)	10.3 (11.9)	31 (25.8%)	8.0 (10.1)	14 (35.9%)	15.6 (14.1)
Physiotherapist, visits	113 (72.0%)	51.6 (54.7)	86 (72.3%)	47.3 (54.2)	27 (71.1%)	65.5 (54.9)
Speech therapist, visits	10 (6.7%)	14.4 (19.4)	4 (3.5%)	5.8 (3.9)	6 (17.1%)	20.2 (23.8)
Home care, weeks	47 (28.8%)	27.2 (31.9)	39 (31.7%)	26.2 (32.7)	8 (20.0%)	32.3 (29.0)
Return to work, weeks ^d^		21.0 (22.7)		17.5 (19.1)		34.4 (30.3)

HEMS: helicopter emergency medical services, ICU: intensive care unit, SD: standard deviation.

^a^ Describes mean (SD) number of days/visits/weeks for patients that used the health care service.

^d^ Productivity loss is based on the working age population (18–67 years). N = 3 patients who did not return to work within 2 years, were included with the maximum of 104 weeks before return to work.

^e^ Comparing ISS 16–24 to ISS≥25, Mann-Whitney U test.

Missing values: 27 (15.5%) missing values for day treatment at a rehabilitation center, 11 (6.3%) missing values for general practitioner visits, 11 (6.3%) missing values for occupational health physician/therapist visits, 15 (8.6%) missing values for psychologist visits, 17 (9.8%) missing values for physio therapist visits, 25 (14.4%) missing values for speech therapist visits, 11 (6.3%) missing values for home care.

### Health care and productivity costs

The total mean costs per patient were €24,760, with mean in-hospital costs of €11,930 and mean post-hospital costs of €7,770 (Table 3; S2 Table: **includes sample size and range**). Both in-hospital and post-hospital costs were twice as high for patients with ISS≥25 compared to patients with ISS 16–24. Mean productivity costs per patient were €8,800. The mean costs of staying at a hospital ward were €7,620. The costs of stay at a rehabilitation centre and day treatments at a rehabilitation centre were three times higher for patients with an ISS≥25, respectively €5,360 and €4,610, compared to mean costs of €1,190 and €1,500 for patients with ISS 16–24.

**Table 3:** Mean (SD) costs by category of service and total health care and productivity costs of adult trauma patients (ISS≥16).

	Total (n = 174)	ISS 16–24 (n = 131)	ISS≥25 (n = 43)
Costs of services	Mean (SD)	Mean (SD)	Mean (SD)
Transport	€1460 (1900)	€1180 (1610)	€2320 (2385)
Stay at a hospital ward	€7620 (10200)	€5720 (6970)	€13570 (15320)
Stay at the ICU	€2360 (1940)	€1810 (1740)	€4030 (1540)
Diagnostics	€1170 (1030)	€1130 (1030)	€1490 (1080)
Stay at a nursing home	€510 (2560)	€650 (2880)	€0 (0)
Stay at a rehabilitation centre	€2090 (7080)	€1190 (4330)	€5360 (12420)
Day treatment at a rehabilitation centre	€2220 (5370)	€1500 (4280)	€4610 (7680)
Physiotherapist	€1220 (1750)	€1120 (1710)	€1500 (1850)
General practitioner	€90 (120)	€80 (110)	€140 (160)
Occupational health care	€180 (330)	€140 (270)	€290 (450)
Psychologist	€240 (600)	€190 (560)	€410 (700)
Speech therapist	€30 (180)	€10 (40)	€110 (370)
Home care	€2080 (7750)	€1690 (5590)	€3270 (12250)
**Costs**			
In-hospital costs	€11930 (11680)	€9180 (8020)	€20290 (16350)
Post-hospital costs	€7770 (13640)	€6030 (10620)	€13300 (19550)
Productivity costs ^a^	€8800 (8420)	€8810 (8110)	€8780 (9500)
Total costs	€24760 (23080)	€20390 (17930)	€38070 (30960)

ISS = injury severity score; SD = standard deviation, ICU = intensive care unit.

^a^ Productivity costs are based on the working age population (18–67 years).

**Fig 1:**
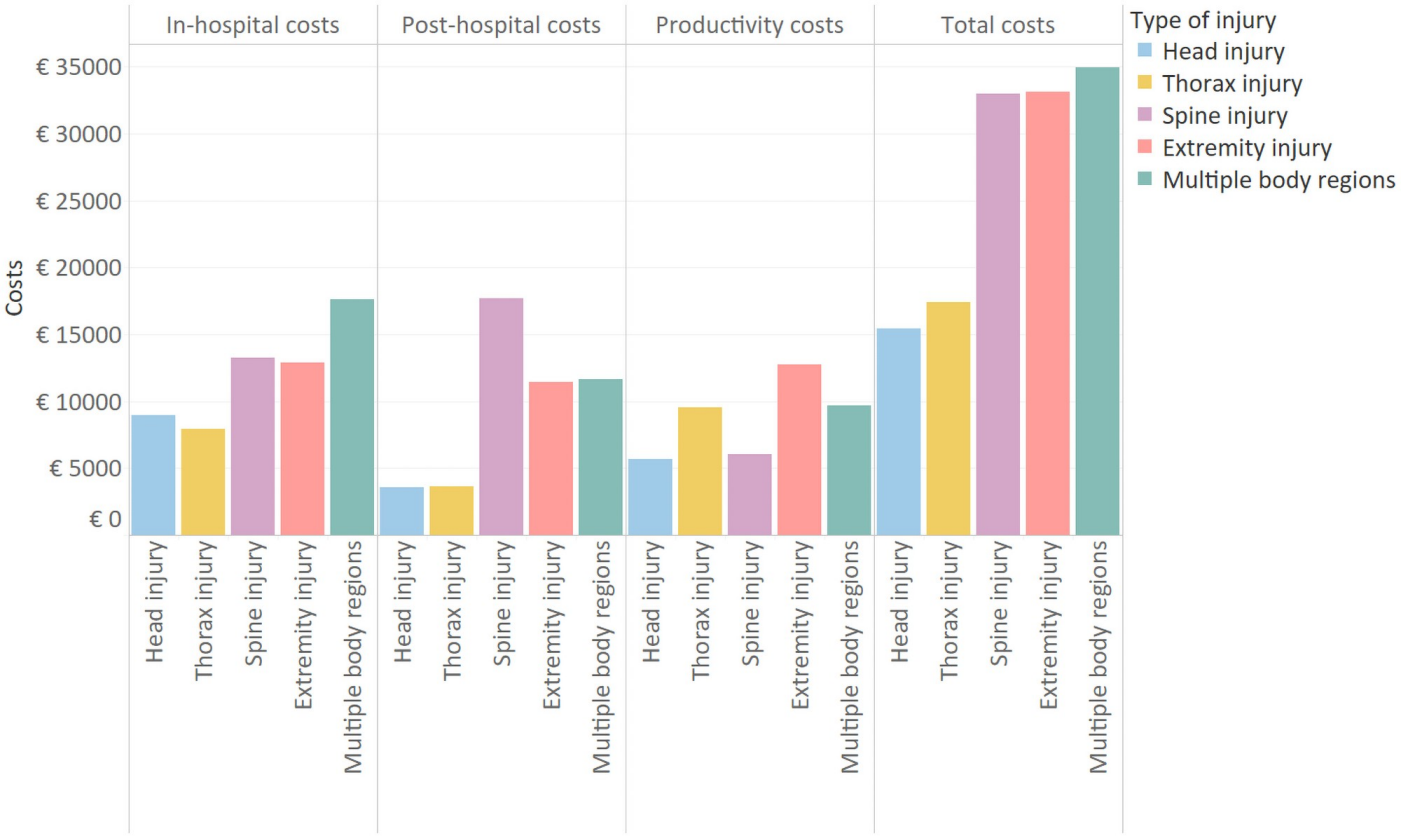
Mean health care and productivity costs by injured body region.

Mean total costs were lowest for patients with head injury (€15,420) and highest for patients with injuries in multiple body regions (€34,960) (Fig 1). Post-hospital costs were highest for patients with spine injuries (€17,700), and productivity costs were highest for patients with extremity injuries (€12,780).

**Fig 2:**
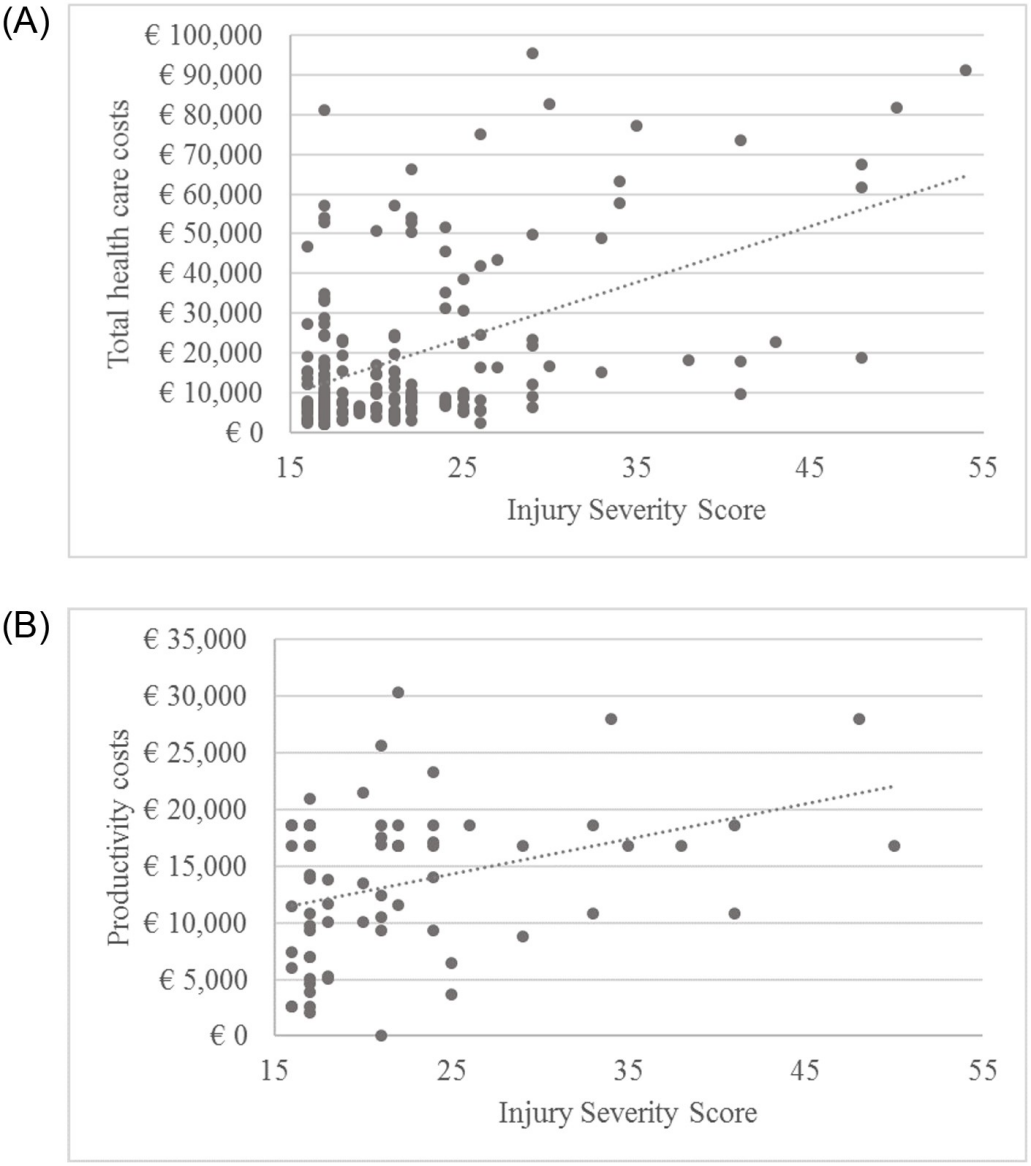
A. Association between injury severity score and total health care costs. B. Association between injury severity score and productivity costs based n = 67 with paid employment and data on productivity loss costs.

In general, patients with ISS ≥25 were on average 10 years younger than patients with ISS 16–24, respectively 47.3 (SD 17.6) years and 56.7 (SD 19.0) years. More than 50% of patients with ISS ≥25 (n = 23) had injuries in multiple body regions, compared to 20% of patients with ISS 16–24. A lot of variation can be observed between patients both for health care and productivity costs (Fig 2A and 2B). Of the patients with ISS 16–24 (n = 139, 75.5%), 11 (7.9%) had health care costs higher than €50,000. These patients had a mean hospital stay of 31.4 days compared to 10.2 days for the patients with ISS 16–24 with costs below €50,000. The patients with ISS 16–24, with high total health care costs also had a higher proportion of severe (AIS≥3) spine (45.5%) and extremity (36.4%) injuries. Of patients with ISS ≥25 (n = 45, 24.5%), 11 (24.4%) had health care costs higher than €50,000 with a mean of 33.2 days at the hospital, compared to 15.3 days for patients with ISS ≥25 and health care costs lower than €50,000. Of the patients with health care costs higher than €50,000, 90.9% had injuries in multiple body regions compared to 58.8% of the patients with ISS≥25 and health care costs lower than €50,000. In patients with ISS ≥25, extremity injuries were more prevalent for those patients with health care costs above €50,000 (63.6% compared to 14.7%). Productivity costs also varied remarkably between patients. Of the patients with known productivity costs (n = 67), 72.1% were male and of the patients with productivity costs higher than €15,000, 96.9% were male.

Lower health status, sex, higher ISS, extremity injury, spinal cord injury and having injuries in multiple bodies regions, showed significant associations with health care costs in the univariable model (Table 4). In the multivariable model, higher ISS, spinal cord injury and lower health status 1 week after injury were associated with higher health care costs.

**Table 4:** Associations with total health care costs and productivity costs based on generalized linear models.

	Health care costs		Productivity costs	
	N = 143		N = 57	
	Exp[β] (95% CI)		Exp[β] (95% CI)	
	Univariable	Multivariable	Univariable	Multivariable
**Age (years)**				
18–44	Ref	Ref	Ref	Ref
45–67	0.96 (0.67–1.36)	1.00 (0.74–1.34)	0.90 (0.67–1.21)	0.89 (0.67–1.18)
≥68	0.70 (0.46–1.07)	1.08 (0.74–1.58)	NA	NA
**Sex: Female**	1.09 (0.80–1.49)	1.16 (0.89–1.50)	0.59 (0.44–0.78)	0.65 (0.49–0.88)
**ISS**	1.05 (1.03–1.08)	1.05 (1.03–1.08)	1.02 (1.00–1.04)	1.02 (0.99–1.04)
**Injured body region (ref: Head)**				
**Extremity**	1.77 (1.13–2.76)	1.43 (0.93–2.19)	1.70 (1.14–2.54)	1.45 (0.96–2.21)
**Thorax**	0.96 (0.66–1.39)	0.96 (0.69–1.34)	1.31 (0.93–1.85)	1.28 (0.92–1.78)
**Spinal cord**	2.27 (1.25–4.11)	2.17 (1.25–3.76)	1.46 (0.79–2.71)	1.24 (0.67–2.29)
**Multiple body regions**	2.27 (1.48–3.27)	1.11 (0.77–1.62)	1.93 (1.32–2.82)	1.40 (0.91–2.14)
**Health status 1 week after injury**^a^	0.26 (0.17–0.39)	0.42 (0.28–0.65)	0.59 (0.44–0.78)	0.93 (0.57–1.53)
**Comorbidities (ref: no comorbidities)**	0.96 (0.71–1.29)	1.11 (0.86–1.43)	0.91 (0.68–1.23)	0.98 (0.75–1.27)

Ref = reference category of categorical variable, CI = Confidence Interval, ISS = Injury Severity Score, NA = Not Applicable.

^a^ Measured with EQ-5D-3L utility score. If patients did not fill in the 1 week questionnaire, the score from 1 month questionnaire was used.

Male sex, higher ISS, having injuries in multiple body regions, having extremity injuries and lower health status were found to be associated with higher productivity costs in the univariable model. In the multivariable model, only male sex was associated with higher costs with lower productivity costs for females.

## Discussion

This study estimated the health care and productivity costs of severely injured patients up to two years after injury. The mean total costs of these patients were €24,760. Length of hospital stay was a key cost driver of total costs. Post-hospital costs where twice as high for patients with ISS ≥25 compared to patients with ISS 16–24, mainly due to costs of stay and day treatment at a rehabilitation centre. Higher ISS, spinal cord injury and lower health status at 1 week after injury were found to be associated with higher health care costs. Although the small sample size, male sex was found to be associated with higher productivity costs. On average, patients with paid employment returned to work 21 weeks after injury. The associated productivity costs had a considerable impact on the total costs. The present study showed that there was a large variety in health care utilization and health care and productivity costs between severely injured patients.

Several other studies provided information on the cost of injury for the entire trauma population [23, 24, 42]. Overall, mean costs of the severe injury population were nearly twice as high compared to mean costs in the entire trauma population, showing an increase in costs with injury severity [23]. A systematic review on acute costs of severe adult polytrauma reported costs of ICU and hospital care up to €76,474 and a systematic review in high income countries found median in-hospital costs of $22,448 [18, 43]. Compared to these studies, in-hospital costs were lower in our population. This may be explained by the predominantly less injured polytrauma patient cohort compared to other studies that reported on severe trauma [18, 44, 45]. This is also reflected by the lower hospital and ICU length of stay. Additionally, this study did not include costs of surgical interventions. This may have resulted in lower mean in-hospital costs compared to previous studies in major trauma patient cohorts. While several studies reported on the in-hospital costs of severe trauma, the consequential costs such as rehabilitation and productivity loss are often overlooked. For decision makers it is important to have a comprehensive view on trauma costs. Our study showed that these costs comprised of approximately 50% of the total costs. Patients with spinal cord or extremity injuries reported the highest mean costs. These results underline the high in-hospital costs related to long hospital stays, especially for those patients. Additionally, post-hospital care, reflecting rehabilitation care were also considerably higher compared to head and thorax injury.

Several longitudinal studies of the health status of severely injured patients showed a high level of functional limitation after 12–36 months. These studies also showed that recovery patterns varied widely between patients groups [7, 15–17, 46, 47]. A previous study in the same severe injury population showed that after twelve months, some patients still reported a lower quality of life compared to the Dutch reference population [15]. From an economic perspective, we found that resource utilization and associated costs also varied widely between severely injured patients. For example, some patients with ISS 16–24 had long hospital stay and high overall costs. These patients had more often spine or extremity injuries. These injuries are also found to be associated with lower post-injury health status [9, 15, 48]. These studies also indicated that the quality of life does not solely depend on injured body area or severity of injury. This is echoed by the findings of our study, which showed that health care utilization is also not solely dependent on injury severity.

A strength of this study was the consideration of long-term care and productivity loss in addition to in-hospital care. Therefore, this study presented a comprehensive overview of both the health care utilization and related costs for the severe injury population. Detailed information was collected at several different times and a combination of registry data and questionnaires was used.

There are several limitations to this study. First, the relatively low inclusion rate of 40.2% reduces the generalizability of the study. Patients with a full recovery are probably more likely to have been lost to follow-up. The differences between participants and non-participants indicate that younger patients and patients with a higher injury severity were more likely to participate, suggesting a non-response bias. This could result in an overestimation of health care and productivity costs However, compared to other studies, our population was older and represented predominantly a less injured polytrauma patient cohort. Second, not all respondents filled out every questionnaire, resulting in missing data on health care utilization. Health care utilization and associated costs per service were based on the respondents with available data. Additionally, data on diagnostics were only available for a sub-group of the population. Finally, the sample sizes in our analysis were small, limiting the statistical power. Future research is needed to further explore the possible predictors of high health care and productivity costs in the severe injury population.

To conclude, health care costs were increasing with injury severity and were especially high for those with spinal cord injury, extremity injury or multiple injuries. Additionally, there is high variety in health care utilization and associated costs in the severe trauma population. Only a part of the severe trauma population need long-term health care including rehabilitation and future research should further investigate which patients are at risk for a slow recovery and use of long-term care. It is important for decision-makers to consider not only in-hospital health care utilization but also the long-term consequences and associated costs related to rehabilitation and productivity loss, as post-hospital care and productivity costs comprise approximately 50% of total costs.

## Supporting information

S1 Table Unit costs (2017 €). (DOCX)

S2 Table Mean and median health care costs and productivity costs in 2017 euro; including range and number of respondents. (DOCX)
