# Supplementary material for: Long-term medical and productivity costs of severe trauma: Results from a prospective cohort study
Source: PLoS One. 2021 Jun 4;16(6):e0252673. doi: 10.1371/journal.pone.0252673 (PMC8177462; doi:10.1371/journal.pone.0252673)
Supplement: S1 Table — (DOCX) [file pone.0252673.s001.docx]

S1 Table. Unit costs (2017 € )

| Unit costs | € |
| --- | --- |
| Transport |  |
| *Self-transport, per ride* | 4.43 |
| *Ambulance transport , per ride* | 690 |
| *Ambulance transport with support of air medical services, per ride* | 5831 |
| *Ambulance transport with support of ground medical services, per ride* | 5831 |
| *Trauma helicopter transport, per ride* | 5281 |
| Hospital |  |
| *Emergency department, per visit* | 265 |
| *In-hospital day generic hospital, per day* | 453 |
| *Intensive Care Unit, per day* | 1213 |
| *Day-treatment hospital, per day* | 282 |
| *Outpatient clinic general hospital, per visit* | 82 |
| Long term care, admission |  |
| *Nursing home, per day* | 172 |
| *Rehabilitation, per day* | 471 |
| *Psychiatric institution, per day* | 309 |
| Long term care, day care |  |
| *Nursing home, per day* | 69 |
| *Rehabilitation, per hour* | 157 |
| *Psychiatric institution, per day* | 100 |
| Home care |  |
| *Domestic care, per hour* | 22 |
| *Help with all day activities, per hour* | 55 |
| *Nursing, per hour* | 75 |
| Healthcare provider |  |
| *General practitioner (GP), per visit* | 34 |
| *Company doctor, per visit* | 53 |
| *Psychologist, per visit* | 65 |
| *Social worker, per visit* | 67 |
| *Physiotherapist, per visit* | 34 |
| *Occupational therapist, per visit* | 34 |
| *Speech therapist, per visit* | 31 |
| *Dietician, per visit* | 21 |
| Productivity |  |
| *Paid labor (male)* | 38.78 |
| *Paid labor (female)* | 32.33 |
