# Supplementary material for: Long-term medical and productivity costs of severe trauma: Results from a prospective cohort study
Source: PLoS One. 2021 Jun 4;16(6):e0252673. doi: 10.1371/journal.pone.0252673 (PMC8177462; doi:10.1371/journal.pone.0252673)
Supplement: S2 Table — (DOCX) [file pone.0252673.s002.docx]

S2 Table. Mean and median health care costs and productivity costs in 2017 euro; including range and number of respondents

| **Costs of services €** | **Total (n=174)** | **ISS 16-24 (n=131)** | **ISS≥25 (n=43))** |
| --- | --- | --- | --- |
| **Transport** |  |  |  |
| Mean (SD) | 1460 (1900) | 1180 (1610) | 2320 (2385) |
| Median (IQR) | 690 (690-690) | 690 (690-690) | 690 (690-5830) |
| Range | 0-5830 | 0-5830 | 0-5830 |
| n | 174 | 131 | 43 |
| **Stay at a hospital ward** |  |  |  |
| Mean (SD) | 7620 (10200) | 5720 (6970) | 13570 (15320) |
| Median (IQR) | 4080 (1810-7930) | 3620 (1810-6340) | 6800 (2940-18570) |
| Range | 450-62970 | 450-46660 | 1260-62970 |
| n | 169 | 128 | 43 |
| **Stay at the ICU** |  |  |  |
| Mean (SD) | 2360 (1940) | 1810 (1740) | 4030 (1540) |
| Median (IQR) | 2430 (0-3640) | 2430 (0-3640) | 3640 (3640-4850) |
| Range | 0-6070 | 0-6070 | 0-6070 |
| n | 184 | 131 | 43 |
| **Diagnostics** |  |  |  |
| Mean (SD) | 1170 (1030) | 1130 (1030) | 1490 (1080) |
| Median (IQR) | 930 (430-1530) | 920 (400-1520) | 1160 (530-2630) |
| Range | 40-5210 | 40-5210 | 260-2630 |
| n | 43 | 38 | 5 |
| **Stay at a nursing home** |  |  |  |
| Mean (SD) | 510 (2560) | 650 (2880) | 0 (0) |
| Median (IQR) | 0 (0-0) | 0 (0-0) | 0 (0-0) |
| Range | 0-23560 | 0-23560 | 0-0 |
| n | 134 | 105 | 29 |
| **Stay at a rehabilitation centre** |  |  |  |
| Mean (SD) | 2090 (7080) | 1190 (4330) | 5360 (12420) |
| Median (IQR) | 0 (0-0) | 0 (0-0) | 0 (0-0) |
| Range | 0-42390 | 0-25910 | 0-42390 |
| n | 134 | 105 | 29 |
| **Day treatment at a rehabilitation centre** |  |  |  |
| Mean (SD) | 2220 (5370) | 1500 (4280) | 4610 (7680) |
| Median (IQR) | 0 (0-0) | 0 (0-0) | 0 (0-8010) |
| Range | 0-34850 | 0-22610 | 0-34850 |
| n | 151 | 117 | 34 |
| **Physiotherapist** |  |  |  |
| Mean (SD) | 1220 (1750) | 1120 (1710) | 1500 (1850) |
| Median (IQR) | 410 (0-1560) | 340 (0-1500) | 680 (0-2270) |
| Range | 0-9690 | 0-9690 | 0-6150 |
| n | 163 | 123 | 40 |
| **General practitioner** |  |  |  |
| Mean (SD) | 90 (120) | 80 (110) | 140 (160) |
| Median (IQR) | 30 (0-140) | 30 (0-100) | 100 (10-230) |
| Range | 0-680 | 0-610 | 0-680 |
| n | 163 | 123 | 40 |
| **Occupational health care** |  |  |  |
| Mean (SD) | 180 (330) | 140 (270) | 290 (450) |
| Median (IQR) | 50 (0-170) | 0 (0-160) | 70 (0-400) |
| Range | 0-2060 | 0-1740 | 0-2060 |
| n | 163 | 123 | 40 |
| **Psychologist** |  |  |  |
| Mean (SD) | 240 (600) | 190 (560) | 410 (700) |
| Median (IQR) | 0 (0-200) | 0 (0-70) | 0 (0-440) |
| Range | 0-3580 | 0-3580 | 0-2600 |
| n | 163 | 123 | 40 |
| **Speech therapist** |  |  |  |
| Mean (SD) | 30 (180) | 10 (40) | 110 (370) |
| Median (IQR) | 0 (0-0) | 0 (0-0) | (0-0) |
| Range | 0-1710 | 0-310 | 0-1710 |
| n | 152 | 117 | 35 |
| **Home care** |  |  |  |
| Mean (SD) | 2080 (7750) | 1690 (5590) | 3270 (12250) |
| Median (IQR) | 0 (0-550) | 0 (0-620) | 0 (0-0) |
| Range | 0-63830 | 0-43620 | 0-63830 |
| n | 163 | 123 | 40 |
| **Costs €** |  |  |  |
| **In-hospital costs** |  |  |  |
| Mean (SD) | 11930 (11680) | 9180 (8020) | 20290 (16350) |
| Median (IQR) | 7570 (4940-13960) | 6800 (4590-10240) | 14110 (8220-30730) |
| Range | 1410-70740 | 1410-51250 | 2340-70740 |
| n | 174 | 131 | 43 |
| **Post-hospital costs** |  |  |  |
| Mean (SD) | 7770 (13640) | 6030 (10620) | 13300 (19550) |
| Median (IQR) | 1460 (330-8640) | 1320 (280-6300) | 3190 (450-18170) |
| Range | 0-69980 | 0-53710 | 0-69980 |
| n | 169 | 128 | 41 |
| **Productivity costs** |  |  |  |
| Mean (SD) | 8800 (8420) | 8810 (8110) | 8780 (9500) |
| Median (IQR) | 8070 (0-16760) | 9300 (0-16760) | 6480 (0-16760) |
| Range | 0-30260 | 0-30260 | 0-27940 |
| n | 104 | 79 | 25 |
| **Total costs** |  |  |  |
| Mean (SD) | 24760 (23080) | 20390 (17930) | 38070 (30960) |
| Median (IQR) | 16670 (7890-33190) | 14860 (6610-27410) | 25850 (12440-67400) |
| Range | 1970-98560 | 1970-81120 | 2440-98560 |
| n | 174 | 131 | 43 |
